# Cognitive screening tools in multiple sclerosis revisited: sensitivity and specificity of a short version of Rao’s Brief Repeatable Battery

**DOI:** 10.1186/s12883-015-0497-8

**Published:** 2015-11-26

**Authors:** Sascha Hansen, Jana Muenssinger, Simona Kronhofmann, Stefan Lautenbacher, Patrick Oschmann, Philipp M Keune

**Affiliations:** Department of Neurology, Klinikum Bayreuth GmbH, Betriebsstätte Hohe Warte, Neurologische Klinik, Sekretariat Prof. Dr. Oschmann, z.Hd. Sascha Hansen, Hohe Warte 8, DE-95445 Bayreuth, Germany; Department of Physiological Psychology, Otto-Friedrich-University Bamberg, Bamberg, Germany

**Keywords:** Cognitive impairment, Multiple sclerosis (MS), Screening, Brief repeatable battery (BRB)

## Abstract

**Background:**

Cognitive deficits are common in multiple sclerosis (MS) and require continuous monitoring. In routine examinations, screening instruments such as the Brief Repeatable Battery (BRB) may serve this purpose. It was suggested that even a shortened version of the BRB, comprising the Symbol Digit Modalities Test (SDMT), Paced Auditory Serial Addition Test (PASAT) and Selective Reminding Test (SRT), may be feasible.

However, an evaluation of sensitivity and specificity of the short BRB in comparison to an independent battery of established tests has not yet occurred. Therefore in the current study, this short version of the BRB was matched against the gold standard of an extensive test battery comprehensively assessing neuropsychological functions.

**Methods:**

127 MS-patients were tested with a short version of the BRB and an extensive procedure. The latter served as the gold standard for defining sensitivity and specificity.

**Results:**

For subtests of the short BRB, estimates of sensitivity (38-44 %) and specificity (81-94 %) were obtained. Combining subtests into a single indicator of cognitive deficits yielded increased sensitivity (78 %), while reducing specificity (65 %).

**Conclusion:**

The short BRB is reasonably sensitive and specific in detecting cognitive deficits. However, these qualities only emerge, if the short BRB is administered as a whole, whereas sensitivity is considerably lower than suggested by previous work, when relying on subtests separately (SDMT, PASAT, SRT). While the short BRB may not be regarded as conclusive as an extensive test battery, it represents a valid and economic screening instrument.

**Electronic supplementary material:**

The online version of this article (doi:10.1186/s12883-015-0497-8) contains supplementary material, which is available to authorized users.

## Background

Neuropsychological deficits occur in about 40-65 % of patients diagnosed with multiple sclerosis (MS). Deficits in attention and information-processing speed as well as long-term and working memory are most common, [1] whereas language and general intellectual ability seem to be largely unaffected [2].

Although these deficits exert a profound impact on patients’ quality of life, they frequently remain undiscovered during routine clinical examinations [3, 4]. This may be attributed to the fact that in clinical practice, time for exhaustive neuropsychological assessments is sparse. In the past, this problem has been acknowledged and tackled by employing short test batteries with the explicit purpose of diagnosing cognitive deficits in MS [5, 6]. Among them, the Brief International Cognitive Assessment for Multiple Sclerosis (BICAMS) [6] and the Brief Repeatable Battery of Neuropsychological Tests (BRB) [7, 8] have been widely accepted as valid screening tools for testing MS-patients [6, 9–11]. The BICAMS is extremely short, taking up only approximately 15 minutes of testing time, while the BRB entails a relatively lengthy testing procedure of approximately 90 minutes duration. However in the BICAMS, there is a decisive lack of assessment of executive functions, since it focuses on processing speed as well as verbal and nonverbal memory [6].

In a pioneering study by Portaccio et al., the performance of a shortened version of the BRB as a quick and economic screening tool was assessed [12]. This short form of the BRB comprises the Paced Auditory Serial Addition Test (PASAT), addressing working memory and attention, the Symbol Digit Modalities Test (SDMT), referring to attention and information processing speed, as well as the Selective Reminding Test (SRT) assessing verbal memory. The authors report that failure on one of these three subtests predicted neuropsychological deficits with high sensitivity (94 %) and specificity (84 %).

While the latter findings are promising concerning the application as a brief assessment tool, they need to be interpreted in the context of a noteworthy limitation. In particular, the authors examined sensitivity and specificity of the aforementioned subtests with regards to cognitive deficits, as determined by the whole BRB and an additional Stroop Test for additional information regarding potential executive dysfunction. Consequently, there was a considerable overlap of tests included in the short screening version of the BRB on the one hand (PASAT, SDMT, SRT), and the procedure which was implemented to derive reliable information about the actual presence of cognitive deficits on the other hand (BRB and Stroop Test). One may argue that similar classification patterns between the shortened version of the BRB and the extended procedure (BRB and Stroop Test) may have been confounded by the fact that all tests of the short version of the BRB were actually included in the more extensive procedure. By this reasoning, estimates of sensitivity and specificity might have been distorted.

The purpose of the current study was to reassess the findings of the pioneering study by Portaccio et al. [12] while avoiding its methodological bias. Thus, a more economic neuropsychological testing of MS patients during clinical routine could be achieved. To this end, sensitivity and specificity of the short version of the BRB were examined with regards to the presence or absence of cognitive deficits as determined by an extensive neuropsychological diagnostic procedure not including the BRB subtests in question. The latter procedure of two hours duration was implemented to thoroughly examine all cognitive domains which may be found deficient in MS-patients. The use of further tests in the screening-procedure (including the Stroop Test) for validation – although desirable – would have significantly hampered the aim of keeping the screening short and was therefore relinquished.

## Methods

### Subjects

A group of 127 patients diagnosed with MS was recruited in the Department of Neurology, Klinikum Bayreuth GmbH, Germany, during the routine clinical process. Data collection was planned before the screening and extensive testing procedure were executed. Recruitment took place from August 2012 to February 2014.Inclusion criteria involved an MS diagnosis according to McDonald criteria [13] and an age range between 18-75 years. Patients were not eligible for study entry if they had severe motor or visual impairments that interfered with handling test material. Demographics and information about clinical characteristics were extracted from patients’ files held by the Department of Neurology, as displayed in Table 1. Participation was voluntary, and all participants provided written informed consent prior to study entry. The study was approved by the ethics committee of the University of Bayreuth.

**Table 1:** Demographical and clinical characteristics of the sample

	CIS	RR-MS	SP-MS	PP-MS	Whole sample
N (%)	11 (8.7)	85 (66.9)	29 (22.8)	2 (1.6)	127 (100)
Age					
Mean	41.6	40.7	50.3	55.0	42.9
SD	9.6	10.3	8.9	28.3	11.0
Min	26	18	28	35	18
Max	54	62	69	75	75
Female sex (%)	8 (72.7)	53 (62.4)	23 (79.3)	1 (50.0)	85 (66.9)
Education					
0-9 years (%)	3 (27.3)	32 (37.6)	13 (44.8)	0 (0.0)	48 (37.8)
10-12 years (%)	4 (36.4)	22 (25.9)	11 (37.9)	2 (100)	39 (30.7)
13+ years (%)	4 (36.4)	31 (36.5)	5 (17.2)	0 (0.0)	40 (31.5)
EDSS					
Median	1	2	4	4	2.5
SD	1.5	1.2	1.3	0	1.8
Min	0	0	4	4	0
Max	4	6	8.5	4	8.5
Disease duration					
Mean	4.01	7.77	16.97	9.6	9.56
SD	4.64	6.91	12.03	7.64	9.2
Min	0	0	1.7	4.2	0
Max	13	35.7	49.7	15	49.7

EDSS = Expanded Disability Status Scale, SD = Standard Deviation

### Procedure

All tests were administered in a standardized individual setting during the routine clinical process. Patients were initially tested with the screening tool, i.e. the short version of the BRB, [12] which took approximately 30 minutes. Testing was conducted by highly trained psychologists specialized in neuropsychology, who could access patients files and clinical information. The procedure comprised the short screening subtests devised by Portaccio et al. [12], namely the Selective Reminding Test (SRT), Symbol Digit Modalities Test (SDMT) and Paced Auditory Serial Addition Test (PASAT).

Subsequently, a comprehensive diagnostic procedure was executed, which was implemented to derive reliable information about the actual presence of cognitive deficits. This was scheduled to take place at the patient's subsequent routine clinical examination (Mean = 4.2 months, SD = 4.2 months). In this context, an extensive neuropsychological test battery was administered. The comprehensive diagnostic procedure had a duration of approximately two hours.

### Measures

#### Screening tool

Following suggestions of Portaccio et al. [12], the short form of the BRB included the SRT for the assessment of declarative episodic long-term memory. Initially, a 12-word list was read to the patient who was required to recall as many words as possible. Afterwards, the clinician reread the words missed by the patient, who had to try and recall the complete list again. This procedure was repeated for a maximum of six trials. The test yields two parameters, i.e. long-term storage (LTS) and consistent long-term retrieval (CLTR). For further information on these parameters, see Additional file 1: Supplement 1.

As a further component, the SDMT [14], assessing information processing speed and attention was included. Patients were required to verbally pair numbers and symbols according to a fixed pattern, the outcome score being the amount of pairings solved correctly within 90 seconds.

Finally the PASAT [15] as a measure of working memory and attention in its three-second interstimulus-interval (ISI) version was applied. Single-digit numbers are read to the patient from tape. The patient has to add each number to the one immediately prior to it. A maximum of 60 correct responses can be achieved. Outcome parameters are listed in Additional file 1: Supplement 2.

#### Extensive neuropsychological test procedure

In addition to the screening tool, an extensive battery of neuropsychological tests was implemented, in order to reliably determine the presence of putative cognitive deficits. The extensive test battery was composed closely adhering to standards set by Benedict [5] as well as Langdon et al. [6]

#### Long-term and working memory

The California Verbal Learning Test (CVLT) [16] was included to assess declarative episodic verbal memory functioning. The paradigm includes five learning-trials of a 16-item word list. Words fall into one of four semantic categories. After the fifth trial, patients were confronted with a distractor-list and a subsequent task to recall items of the initial list. A delayed recall task was implemented after approximately 20 minutes.

To address working memory capacity, two subtests of the Wechsler Memory Scale (WMS-R) [17] were implemented. The first test (digit span forward) required a patient to instantly repeat strings of numbers of increasing length, read out by the examiner. In a variation of this task (digit span backwards) numbers were to be reproduced in reversed order. The second subtest (block span), was a nonverbal equivalent of the digit span task. Patients were required to reproduce several sequences of increasing length on a tapping board (forwards and backwards). It should be noted that particularly the backwards conditions of these tasks address working memory capacity, whereas performance in the forward conditions is also affected by general cognitive processing speed.

#### Attention

Attentional parameters were assessed by means of a standardized, computer-based Test of Attentional Performance (TAP) [18]. Three subtests were used:

#### Alertness

Motor response times were obtained in two conditions. First, patients were required to respond to a cross appearing in the middle of the screen (variable ISI) by pressing a button as quickly as possible, yielding a measure of intrinsic alertness (basic attentional intensity) [19]. In the second condition, the appearance of the cross was preceded by a warning tone. The latter condition assessed the capability to focus attention on an anticipated event (phasic alertness)[19].

#### Go-Nogo

Motor response times were obtained in context of a selective response task, in which patients had to press a button in response to the appearance of a predefined critical stimulus, while a response had to be avoided in case of the appearance of a noncritical stimulus. The test addresses selective attention and response inhibition.

#### Divided Attention

Additional motor response times were assessed during a task in which attention had to be allocated simultaneously to visual and auditory stimuli. Patients were required to press a button as soon as moving crosses on the screen formed a square, and when a sequence of alternating high and low-pitch tones was broken as the same tone appeared twice in sequence. It should be noted that this test also addresses working memory and cognitive flexibility due to the fact that the putative occurrence of response cues during the test (square, same tone twice in a row) needs to be continuously monitored and compared to the memorized response cues, predefined during the test instructions.

#### Verbal and nonverbal fluency

The Regensburger Wortschatz-Test (english: lexical test; RWT) [20] is a test measuring verbal fluency and divergent thinking. Several time-restricted verbal tasks (one-minute) were included. In a first task, patients had to generate as many words as possible, beginning with a specific letter (phonematic fluency). Subsequently, words had to be generated belonging to a specific category (semantic fluency). In addition, patients were required to produce words, switching back and forth between two predefined first letters and between two predefined categories (see Additional file 1: Supplement 1). As such, the latter tasks involved an additional emphasis on working memory functioning.

The Five-Point-Test (FPT) [21] was used for the assessment of nonverbal fluency. Patients were required to draw as many different patterns as possible on a piece of paper by connecting dots within several squares during a three-minute time span. As was the case in the RWT, working memory functioning may be regarded as a crucial element to solve this test as patients had to ensure not to repeat any pattern. Additional information on all test parameters can be reviewed in Additional file 1: Supplement 2.

### Derivation of sensitivity and specificity estimates

Data was processed by means of SPSS 20.0 (IBM). In order to determine sensitivity and specificity of the short BRB, several parameters were derived: Sensitivity describes the ratio of patients identified as cognitively impaired by both the short BRB and the extensive test battery (true positives), whereas specificity is the ratio of patients identified as cognitively unimpaired by the short BRB and the extensive test battery (true negatives). Performance on any given test was regarded as impaired if it involved a percentage rank (PR) < 16, based on age-corrected normative data of each test. Confidence intervals (95 %; CI) were calculated according to an efficient-score method, corrected for continuity [22].

#### Calculation of Parameters

Sensitivity and specificity were derived for several configurations. First and foremost, the whole screening was matched with the whole extensive test battery, a patient being considered impaired if one test parameter indicated cognitive impairment (PR < 16).

Furthermore, sensitivity and specificity were also calculated for each subtest of the BRB (SRT, SDMT, PASAT) in comparison to the whole extensive test battery. Again, a patient was considered impaired if at least one test parameter indicated cognitive impairment (PR < 16).

Finally, the three different cognitive domains addressed by the three subtests of the BRB were considered. The three cognitive domains were conceptualized as memory (SRT), speed (SDMT) and working memory and attention (PASAT). Tests of the extensive procedure were assorted to these domains, as displayed in Table 2. Sensitivity and specificity of a respective BRB subtest in predicting impairment in a corresponding domain of the extensive procedure were then determined. A domain of the extensive procedure was considered impaired if scores in any test belonging to that domain fell below the threshold of PR <16. For further information on test parameters, see Additional file 1: Supplement 2.

**Table 2:** Composition of cognitive domains in the screening and the extensive test battery.

Domain	Equivalent in screening	Equivalent in extensive test battery
Memory	SRT	CVLT
Speed	SDMT	TAP – AL TAP – GO WMS-R-DS forward WMS-R-BS forward
Working memory	PASAT	TAP – GA WMS-R-DS backward WMS-R-BS-backward FPT RWT

SRT: Selective Reminding Test; SDMT: Symbol Digit Modalities Test; PASAT: Paced Auditory Serial Addition Test; CVLT: California Verbal Learning Test; TAP: test of attentional performance; AL: Alertness: GO: Go-NoGo; GA: Divided Attention; WMS-R: Wechsler Memory Scale – Revised; DS: digit span; BS: block span; FPT: Five-Point-Test; RWT: Regensburger Wortschatz Test

## Results

Considering the extensive testing procedure, 72 patients (57.1 %) showed cognitive deficits, whereas in the short version of the BRB, 75 patients (59.5 %) were identified as cognitively impaired. When matching the whole of the screening (SRT, SDMT, PASAT) with the gold standard of the extensive test battery, sensitivity was 77.8 % and specificity was 64.8 % (Table 3).

**Table 3:** Performance of screening-subtests (single and combined)

	Sensitivity (95 % CI)	Specificity (95 % CI)
SRT	38.36 % (27.44 % - 50.51 %)	81.48 % (68.13 % - 90.30 %)
SDMT	43.84 % (32.41 % - 55.91 %)	94.44 % (83.66 % - 98.55 %)
PASAT	41.67 % (30.35 % - 53.88 %)	87.04 % (74.48 % - 94.19 %)
SRT + SDMT + PASAT	77.78 % (66.15 % - 86.39 %)	64.81 % (50.55 % - 76.97 %)

CI: Confidence Interval; SRT: Selective Reminding Test; SDMT: Symbol Digit Modalities Test; PASAT: Paced Auditory Addition Test

Screening subtests were also individually matched with the whole of the extensive test battery (Table 3), resulting in lower sensitivity values (38 % for the SRT, 41.7 % for the PASAT and 43.8 % for the SDMT), but increasing specificity values (81.5 % for the SRT, 87.0 % for the PASAT and 94.4 % for the SDMT).

Finally, obtained estimates of sensitivity and specificity of the three subtests of the screening matched with their respective domains from the extensive test battery are displayed in Table 4. Sensitivity ranged from 49.1 % in the working memory domain (PASAT) to 60.0 % in the memory domain (SRT), and a corresponding estimate in the speed domain of 52.9 % (SDMT). Specificity was considerably higher, ranging from 81.5 % in the memory domain to 89.5 % in the speed domain, with the memory domain falling in between these two with a value of 84.9 %.

**Table 4:** Performance of screening-subtests in respective extensive test battery domains

	Sensitivity (95 % CI)	Specificity (95 % CI)
SRT	60.00 % (42.21 % - 75.65 %)	81.52 % (71.78 % - 88.57 %)
SDMT	52.94 % (38.60 % - 66.84 %)	89.47 % (79.79 % - 95.02 %)
PASAT	49.06 % (35.25 % - 63.00 %)	84.93 % (74.21 % - 91.88 %)

CI: Confidence Interval; SRT: Selective Reminding Test; SDMT: Symbol Digit Modalities Test; PASAT: Paced Auditory Addition Test

In sum, consistent estimates above chance level for both, sensitivity and specificity, were only reached in case of the first approach, i.e. matching the whole of the screening with the extensive test battery. While utilizing individual screening subtests increased specificity, this approach involved considerably attenuated sensitivity. Further information regarding the number of cognitively impaired patients in each subtest of the screening as well as each cognitive domain in the extensive testing procedure can be reviewed in Additional file 1: Supplement 3.

## Discussion

A thorough diagnosis of cognitive deficits in MS-patients is a time- and resource-consuming process consisting of a large number of neuropsychological test procedures. In clinical practice, where time for excessive assessments is sparse, such a thorough diagnosis is often not feasible. Therefore, short assessment methods could essentially improve the diagnostic process.

In the current study, MS-patients completed both, an economic screening session by means of a brief version of the BRB, as well as an extensive diagnostic procedure closely adhering to established standards of an extensive testing in MS^5,6^. The proportion of patients who were identified as displaying cognitive deficits was relatively similar between the screening (59.5 %) and the extensive procedure (57.1 %). As such, it resembles common estimates, according to which neuropsychological deficits occur in about 40-65 % of MS-patients [1].

Matching the whole of the short version of the BRB with the whole extensive testing procedure resulted in a sensitivity of 77.8 % and a specificity of 64.8 %. In the current work, this matching constellation was the only one which produced consistent estimates of sensitivity and specificity above chance level. In contrast, a clearly different pattern of results emerged when BRB subtests were individually matched with results of the entire extensive testing procedure. Here, sensitivity was lower (38.7 % to 43.8 %), whereas specificity was somewhat increased (81.5 % to 94.4 %). Sensitivity estimates for the individual subtests of the short version of the BRB referring to the respective domains of the extensive testing procedure resulted in only marginally increased sensitivity (49.1 %-60.0 %) and approximately equal specificity (81.5 %-89.5 %). While this common pattern of elevated specificity relative to sensitivity on the subtest-level is compatible with previous reports by Portaccio et al. [12], overall estimates of sensitivity and specificity obtained in the current study are considerably lower than those reported by the latter authors.

While Portaccio et al. [12] reported specificity estimates of 84 % for the short version of the BRB, in the current study, 64.8 % were obtained. The same pattern holds for sensitivity, where Portaccio et al. [12] reported 94 %, whereas in the current study, 77.8 % were obtained. As previously indicated, in the study by Portaccio et al., [12] there was a considerable overlap of tests included in the short screening version of the BRB on the one hand, and the procedure which was implemented to derive reliable information about the actual presence of cognitive deficits on the other hand. Similar classification patterns between the shortened version of the BRB and the more extensive procedure in the latter work might hence have been the result of overestimation. In support of this assumption, estimates of the current work, which were based on an extensive diagnostic procedure completely independent from the screening, were considerably lower.

While estimates of specificity and sensitivity in the current work were lower than those of Portaccio et al., [12] it is remarkable that despite the fact that a relatively independent diagnostic procedure was implemented, estimates were still reasonable, when the screening was considered as a global predictor. In particular the important parameter *sensitivity*, representing the proportion of patients adequately identified as cognitively impaired by the screening, showed a reasonable estimate. While adequacy in identifying patients without cognitive deficits, reflected by specificity, was somewhat lower, it may be argued that the latter group of patients would probably be examined by an extensive procedure subsequently by default in context of routine clinical practice. In that case, a false positive result would yield a subsequent extensive examination to verify the screening result. The fact that the screening actually identified *more* patients as cognitively impaired than the extensive testing procedure further underlines its usefulness for a short assessment and its appropriateness as a screening tool. In sum, the current findings may be regarded as complementing previous suggestions by Portaccio et al., [12] as they provide further support for the utility of the short version of the BRB as a valid screening tool. Nevertheless, it needs to be emphasized that according to the current findings, the feasibility is only given when the screening is regarded as a global indicator.

Another noteworthy aspect of the current study concerns the usefulness and necessity of the application of the PASAT in future diagnostic procedures. In the past, it has often been pointed out that the PASAT is somewhat flawed since it requires a certain amount of mathematical ability [23]. It also acts as a potential stressor, since patients are required to keep up with the pace of the number reading [24]. Therefore, the question has been raised whether to completely abstain from using it in test batteries and instead favouring the SDMT, which is generally better accepted by patients [25]. The current study shows that, even though the SDMT is slightly superior to the PASAT in terms of specificity, each test by itself has a fairly low sensitivity. Only combined with each other and the SRT can they reach sufficiently high levels of sensitivity to be deemed as having an adequate predictive value.

In the current study, we decided against implementing a Stroop-Test in addition to the short version of the BRB. On the one hand, executive functions are already being addressed by the PASAT. On the other, a Stroop-Test would significantly lengthen the screening-procedure, [26] which would countermand the aim of an economic screening tool. However, it should be noted that the underlying cognitive constructs of the PASAT are still a matter of debate [23, 24] and that a short test for executive function in MS is still lacking. It is for this last reason that we decided against the BICAMS and for the short BRB in our study. Both approaches require approximately 15 minutes (testing time only) and both consist of three subtests. But even though validation of the BICAMS is currently underway in several countries as a short screening tool in MS [27], we consider the three subtests of the BRB to better cover the width – if not the depth – of neuropsychological constructs possibly affected in MS, since they have the arguable benefit of also assessing executive functions through the PASAT.

While our results are generally compatible with the extant literature and provide an extension with regards to the previous work by Portaccio et al., [12] it should be noted that in the current study, the extensive diagnostic procedure was not implemented on the same assessment occasion as the screening. Consequently, a second appointment had to be scheduled. Relatively decreased specificity and sensitivity estimates may have been affected by these circumstances. On the other hand, it is noteworthy that despite the delay between testing procedures, specificity and sensitivity estimates of the short version of the BRB were still reasonable. However, since stability of cognitive deficits in MS over time is still debated and longitudinal studies in this field of research are scarce, [28] time intervals between screening and extensive testing should be avoided in further research in this area.

## Conclusion

In summary, current results show that the suggested short screening tool consisting of the BRB-subtests PASAT, SDMT and SRT is indeed a valid instrument for a time- and cost-efficient first assessment of cognitive deficits in MS-patients when it is regarded as a global indicator.

## Additional file

Additional file 1:
**Supplement 1.** Additional information on neuropsychological tests. **Supplement 2.** - Additional information on test parameters. **Supplement 3.** – flow diagram on diagnostic procedures and number of cases (DOC 46 kb)
