# Supplementary material for: Cognitive screening tools in multiple sclerosis revisited: sensitivity and specificity of a short version of Rao’s Brief Repeatable Battery
Source: BMC Neurol. 2015 Nov 26;15:246. doi: 10.1186/s12883-015-0497-8 (PMC4660646; doi:10.1186/s12883-015-0497-8)
Supplement: Additional file 1: — Supplement 1. Additional information on neuropsychological tests. Supplement 2. - Additional information on test parameters. Supplement 3. – flow diagram on diagnostic procedures and number of cases (DOC 46 kb) [file 12883_2015_497_MOESM1_ESM.doc]

Supplement 1 - Additional information on neuropsychological tests:

Selective Reminding Test: The SRT involves the presentation of a maximum of six trials of a 12-item word list, in which the examiner only presents those words not recalled on the immediately preceding trial. Presentation is discontinued once a patient recalls all words of the list on two consecutive trials. The test yields two parameters, which are long-term storage (LTS) and consistent long-term-retrieval (CLTR). A word is assumed to enter LTS if it is recalled on two consecutive trials. If it is not reported on consecutive trials, it is then assumed that the patient failed to retrieve it. Thus, CLTR scores words recalled consistently throughout learning trials.

Regensburger Wortschatztest (word fluency test): The following versions of the RWT and their respective norms were employed, depending on a patients previous experience with the test:

- simple semantic trial: Animals OR Foods
- complex semantic trial: Sports and Fruits OR Clothing and Flowers
- simple phonematic trial: S-words OR M-words
- complex phonemativ trial: G-R or H-T

Supplement 2 - Additional information on test parameters:

| **Test** | **Relevant Parameter** | **Mean PR*** |
| --- | --- | --- |
| PASAT | Number of correct responses | 46.3 |
| SDMT | Number of correctly identified items | 41.9 |
| SRT | - LTS: Items recalled on at least two consecutive trials, summed up for all trials. - CLRT: Items recalled on at least two consecutive trials and retained to the last trial, summed up for all trials. | 49.4  45.7 |
| CVLT | - Number of items recalled on initial trial - Number of items recalled over all trials - Number of items recalled after distractor list - Number of items recalled after retention interval | 55.9  55.5  48.9  51.8 |
| WMS-R-Digit Span | - Number of correct repetitions forward - Number of correct repetitions backward | 38.9  45.3 |
| WMS-R-Block Span | - Number of correct repetitions forward - Number of correct repetitions backward | 54.4  49.0 |
| TAP-Alertness | - Reaction time for ‘tonic’ trials - Reaction time for ‘phasic’ trials | 32.2  25.5 |
| TAP-Go/NoGo (2 aus 5) | - Reaction time - Number of omissions - Number of errors | 41.8 |
| TAP-GA | - Reaction time for auditory stimuli - Reaction time for visual stimuli - Number of omissions (auditory and visual) - Number of errors (auditory and visual) | 25.5  41.9 |
| RWT | - Word count on simple semantic fluency - Word count on complex semantic fluency - Word count on simple phonematic fluency - Word count on complex semantic fluency | 46.8  45.5  31.0  33.8 |
| FPT | Number of correct, unique items drawn | 37.5 |

*mean percentage ranks have not been added for variables which are not normally distributed

All abbreviations and detailed information concerning testing procedures can be found in the ‘methods’ section of the article

Supplement 3 – flow diagram on diagnostic procedures and number of cases

N = 127

Screening

cognitive deficits present

N = 75

No cognitve deficits detected

N = 52

PASAT

N = 37

SRT

N = 38

SDMT

N = 35

Extensive testing

cognitive deficits present

N = 72

No cognitve deficits detected

N = 55

working memory domain

N = 53

speed domain

N = 51

memory domain

N = 35
